# Rural–urban disparities in atrial fibrillation–related diagnostic testing and medical procedures in Canada^†^

**DOI:** 10.1093/europace/euag054

**Published:** 2026-04-14

**Authors:** Mohammed Shurrab, William F McIntyre, Christopher C Cheung, Jason G Andrade, Feng Qiu, Olivia Haldenby, Habib R Khan, Ratika Parkash, Jeff S Healey, Dennis T Ko

**Affiliations:** Cardiology Department, Health Sciences North, Northern Ontario School of Medicine University, 41 Ramsey Lake Rd, Sudbury, Ontario, Canada P3E 5J1; Health Sciences North Research Institute, 56 Walford Rd, Sudbury, Ontario, Canada P3E 2H3; Institute of Health Policy, Management and Evaluation, University of Toronto, 155 College Street, Toronto, Ontario, Canada M5T 3M6; ICES, 2075 Bayview Ave, Ontario, Canada M4N 3M5; Division of Cardiology, Department of Medicine, Hamilton Health Sciences, McMaster University, Hamilton, Ontario, Canada; Population Health Research Institute, McMaster University, Hamilton, Ontario, Canada; Schulich Heart Centre, Sunnybrook Health Sciences Centre, University of Toronto, Toronto, Ontario, Canada; Heart Rhythm Services, Department of Medicine, University of British Columbia, Vancouver, British Columbia, Canada; Center for Cardiovascular Innovation, Vancouver, British Columbia, Canada; ICES, 2075 Bayview Ave, Ontario, Canada M4N 3M5; ICES, 2075 Bayview Ave, Ontario, Canada M4N 3M5; London Health Sciences Center, Schulich School of Medical and Dentistry, University of Western Ontario, London, Ontario, Canada; Division of Cardiology, Queen Elizabeth II Health Sciences Center, Dalhousie University, Halifax, Nova Scotia, Canada; Division of Cardiology, Department of Medicine, Hamilton Health Sciences, McMaster University, Hamilton, Ontario, Canada; Population Health Research Institute, McMaster University, Hamilton, Ontario, Canada; Institute of Health Policy, Management and Evaluation, University of Toronto, 155 College Street, Toronto, Ontario, Canada M5T 3M6; ICES, 2075 Bayview Ave, Ontario, Canada M4N 3M5; Schulich Heart Centre, Sunnybrook Health Sciences Centre, University of Toronto, Toronto, Ontario, Canada

**Keywords:** Atrial fibrillation, Disparities, Rural, Universal Health Care

## Introduction

In a universal health care system, geographic disparities in atrial fibrillation (AF) care remain poorly understood. Canada, the second largest country in the world, is also one of the least densely populated (4.2 inhabitants/km^2^ ranking 239/250 nations).^1^ Rural Canadians consistently experience poorer health outcomes.^2^ Our recent work has shown that rural patients with AF had lower uptake of evidence-based therapies and higher rates of acute care utilization.^3^ Despite universal health care coverage, rural–urban disparities in AF outcomes persist, representing a major health care challenge in Canada, where AF affects approximately 3% of the population and accounts for an estimated $2 billion in annual health care costs.^4,5^

Consensus recommendations advise baseline cardiac testing for all patients with AF and consideration of rhythm-control strategies.^4^ However, whether access to these recommended investigations and procedures differs by rural vs. urban residence remains unclear. Accordingly, this study evaluated rural–urban differences in the use of AF-related diagnostic tests and medical procedures among patients presenting to the emergency department (ED) with AF in Ontario, Canada.

## Methods

We conducted a population-based retrospective cohort study by linking multiple administrative databases in Ontario. The cohort included adults aged ≥18 years presenting to the ED with a primary diagnosis of AF between 1 April 2012 and 31 March 2022. AF was identified using a previously validated algorithm.^6^ We included only AF diagnoses recorded at ED presentation. The primary exposure was rural residence, defined as living in a community with a population of ≤10,000, consistent with the definition from Statistics Canada Postal Code.^7^ Outcomes included AF-related diagnostic testing [electrocardiography (ECG), echocardiography, ambulatory ECG monitoring, and stress testing] and medical procedures, including rhythm-control strategies (electrical cardioversion or catheter ablation) and pacemaker or defibrillator implantation. We identified ECGs using physician billing and procedural codes occurring after the index ED visit. The ECG outcome did not include the index ED ECG, aiming to evaluate follow-up cardiac testing after ED discharge, rather than the diagnostic ECG performed at presentation. The index date was defined as the ED presentation with AF. Diagnostic tests were assessed only if performed after the index ED visit. Pacemaker and defibrillator implantation were included to capture device-based interventions that may be encountered in AF patients (tachy-brady syndrome or AF-related cardiomyopathy). Patients were followed from the index ED visit until death, loss of provincial health coverage eligibility, or the end of the follow-up period. Cox proportional hazards regression models were used to estimate adjusted hazard ratios (HRs) for each outcome at 100 days and 1 year. Models were adjusted for common comorbidities. Frailty was assessed using the validated Hospital Frailty Risk Score.^8^ All analyses were conducted using SAS version 9.4.

## Results

Among 104 195 patients with AF presenting to the ED, 16 860 (16.2%) resided in rural communities. The mean age was similar between rural and urban patients (69.5 vs. 69.4 years), while females comprised 44.3% of the rural group and 47.7% of the urban group.

Within 1 year of ED presentation, patients with AF living in rural Ontario had significantly lower rates of AF-related diagnostic testing compared with urban patients, including ECG (76.7% vs. 83.3%; HR 0.78, 95% CI 0.77–0.80), echocardiography (65.3% vs. 68.8%; HR 0.90, 95% CI 0.88–0.92), ambulatory ECG monitoring (73.2% vs. 76.1%; HR 0.92, 95% CI 0.90–0.94), and stress testing (25.8% vs. 30.3%; HR 0.80, 95% CI 0.78–0.83) (all *P* < 0.001). Among rural patients, older age, female sex, frailty, higher CHA_2_DS_2_-VASc score, chronic obstructive pulmonary disease, and lower socioeconomic status were associated with lower rates of diagnostic testing.

**Figure 1:**
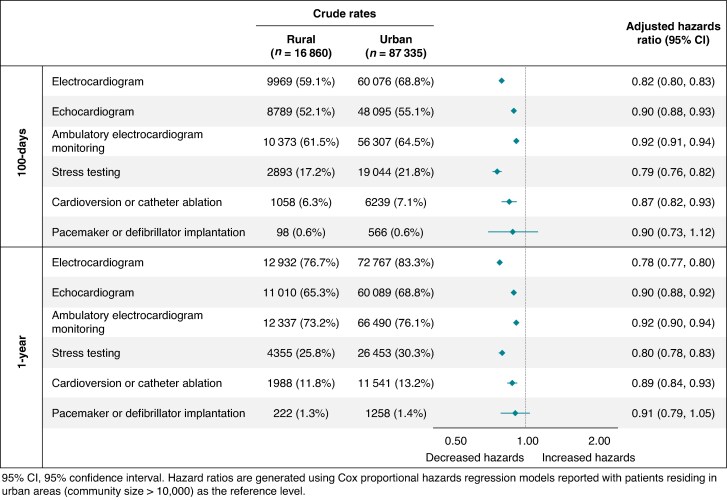
AF-related diagnostic testing and medical procedures.

Within 1 year, rural patients also underwent fewer rhythm-control procedures compared with urban patients (11.8% vs. 13.2%; HR 0.89, 95% CI 0.84–0.93; *P* < 0.001). Older age, female sex, and frailty were associated with lower rates of rhythm-control procedures. Pacemaker or defibrillator implantation rates were similar between rural and urban groups (1.3% vs. 1.4%; *P* = 0.21). Rates of diagnostic testing and medical procedures at 100 days and 1 year are shown in *Figure 1*.

## Discussion

In this large, population-based cohort study, rural residence was associated with lower rates of guideline recommended diagnostic testing, including ECG, echocardiography, ambulatory ECG monitoring, and stress testing, as well as lower use of rhythm-control strategies such as electrical cardioversion or catheter ablation.

In our prior work,^3^ rural patients with AF were less likely to receive cardiology follow-up after ED presentation and experienced higher acute care utilization. In the present study, rates of guideline-recommended testing were significantly lower among rural patients, likely reflecting access barriers, local resource limitations, and workforce shortages. Given the proven benefits of early rhythm control in selected AF populations, including improved cardiovascular outcomes, these differences in procedural intervention may indicate that rural patients have reduced access to certain rhythm-control strategies for optimal disease management.^9,10^ Importantly, lower rates of rhythm-control procedures should not necessarily be interpreted as evidence of inequity. Clinical decision-making regarding antiarrhythmic drug therapy or catheter ablation is highly individualized and may reasonably reflect differences in age, frailty, comorbidity burden, symptom severity, AF subtype, or patient preference.

Potential strategies to mitigate geographic barriers to specialist care may include telecardiology programmes and remote rhythm monitoring technologies, which can facilitate earlier detection, specialist consultation, and management of AF in patients residing in remote or rural regions. These findings may also be relevant to other global settings, including parts of Asia and Africa, where large rural populations and limited access to specialist cardiovascular care may contribute to even greater disparities in the management of AF.

Several limitations of this study should be considered. Residual confounding remains possible, as certain clinical factors including AF subtype, disease severity, referral patterns, and patient preference were not captured in the administrative datasets. Residence was determined at the index ED visit and changes in residence over time were not captured. Death or hospitalization may act as competing risks for diagnostic testing or procedures in this older population, which may influence the estimated associations.

## Conclusions

In this large population-based cohort, substantial rural–urban disparities persist in the delivery of essential AF-related diagnostic testing and interventions despite universal health care coverage.

## Data Availability

The dataset from this study is held securely in coded form at ICES. Although data sharing agreements prohibit ICES from making the data set publicly available, access may be granted to those who meet prespecified criteria for confidential access, available at www.ices.on.ca/DAS. The full dataset creation plan and underlying analytic code are available from the authors on request, understanding that the programmes may rely on coding templates or macros that are unique to ICES.
